# Inflammatory Bowel Disease and Irritable Bowel Syndrome: What Differences in Mentalization Abilities? A Scoping Review

**DOI:** 10.3390/ijerph20237125

**Published:** 2023-11-30

**Authors:** Agata Benfante, Fabio Cisarò, Davide Giuseppe Ribaldone, Lorys Castelli, Nikolas Sandroni, Annunziata Romeo

**Affiliations:** 1 Department of Psychology, University of Turin, 10124 Turin, Italy; lorys.castelli@unito.it (L.C.); nikolas.sandroni@edu.unito.it (N.S.); annunziata.romeo@unito.it (A.R.); 2 Division of Digestive Endoscopy, Città della Salute e della Scienza University-Hospital, 10126 Turin, Italy; fabio.cisaro@gmail.com; 3 Division of Gastroenterology, Città della Salute e della Scienza University-Hospital, 10126 Turin, Italy; davidegiuseppe.ribaldone@unito.it; 4 Department of Medical Sciences, University of Turin, 10126 Turin, Italy

**Keywords:** inflammatory bowel disease, irritable bowel syndrome, mentalization, metacognition, quality of life

## Abstract

Mentalization is a psychological process that enables individuals to understand the self and others in terms of intentional mental states. The aim of this scoping review was to provide an overview of the findings on mentalization in patients with inflammatory bowel disease (IBD) and irritable bowel syndrome (IBS). A literature search, in line with the Preferred Reporting Items for Systematic Review and Meta-analysis Protocols extension for Scoping Review guidelines, was conducted in the following bibliographic databases: PubMed, PsycINFO, and Scopus. Databases were queried using the following strings (with Boolean operators): (“mentaliz*” OR “metacogniti*” OR “theory of mind” OR “ToM” OR “reflective function*”) AND (“irritable bowel syndrome” OR “IBS” OR “inflammatory bowel disease” OR “IBD”). In line with the eligibility criteria, seven articles were included. Results showed that no significant differences in metacognitive ability were found between patients in the IBD and IBS groups. This review revealed the mentalizing difficulties for patients with IBD and IBS. These results should be interpreted with caution since they are based on a few studies that used different instruments to assess mentalizing processes. Future studies are needed to clarify the role of mentalization in patients with these gastrointestinal conditions.

## 1. Introduction

Mentalization is a psychological process that enables an individual to understand, implicitly and explicitly, the self and others in terms of intentional mental states (e.g., feelings, desires, beliefs, attitudes, goals, and needs) [1,2]. Implicit mentalization refers to unconscious and automatic operations of the capacity to imagine mental states, whereas explicit mentalization implies a conscious use of such operations, which occurs, for example, during psychotherapeutic work [3].

This capacity, which develops from early childhood in a secure attachment relationship with the caregiver [1,4,5], allows the individuals an affective and interpersonal understanding of their own and others’ mental states, with consequences for the social dimension of life [6]. Furthermore, an effective ability to mentalize provides support for individuals coping with adversity [7].

The concept of mentalization is connected to and partially overlaps with other constructs derived from different study fields [2,3]. Among these, particular attention should be paid to the concepts of the Theory of Mind (ToM), metacognition, and reflective functioning, which are occasionally used as synonyms in research without considering the peculiarities to which they refer.

Mentalization is conceptually derived from the ToM, which was initially introduced in the context of studies on social cognition [3,6,8,9]. It refers to an individual’s mental capacity useful for appreciating different mental states in others and identifying these states according to their purpose to interpret their behavior [2,3,9].

Otherwise, the concept of metacognition, originally developed in the field of developmental and educational psychology, includes processes that allow individuals to reflect on their thoughts [10]. It covers explicit and conscious areas of mentalization. However, the sense of self and others that results from metacognitive processes is formed through the integration of a wide range of embodied, cognitive, emotional, and interpersonal experiences [11].

Finally, reflective functioning is a synonym for mentalization and is derived from the combination of the psychoanalytic concept and empirical research on attachment [5,12]. This term acquired more specificity when Fonagy and colleagues considered the need to operationalize the construct of mentalization, developing the Reflective Functioning (RF) scale [4,12,13].

The assessment of mentalization has been carried out not only in psychopathological disorders but also in stress regulation and Functional Somatic Disorders (FSDs), which include different syndromes without a clearly established organic cause [2,14].

According to the mentalization-based approach, several biological and environmental factors are known to be predisposing to increased vulnerability to the development of FSDs [2,3,14,15]. If psychological and/or physiological precipitating factors are added to this condition, an individual may experience dysfunction in the stress system (Hypothalamic–Pituitary–Adrenal, or HPA, axis) [2,14,15]. A mentalizing impairment and the dysfunctional use of secondary attachment strategies (i.e., attachment deactivating and hyperactivating strategies), which individuals employ to cope with stress, contribute to maintaining FSD symptoms, further exacerbating stress [1,2,6,14,15].

Among FSDs, significant dysfunctions in the HPA and gut–brain axis have been found in Irritable Bowel Syndrome (IBS) [2,16,17]. IBS is a functional gastrointestinal syndrome characterized by altered bowel habits, abdominal pain, and bloating, with a worldwide prevalence of 4% based on the Rome IV criteria. It is diagnosed with symptom-based criteria since specific tests or biomarkers are not yet available [17,18,19].

Similar HPA and gut–brain axis dysfunctions and symptoms occur in Inflammatory Bowel Disease (IBD), which is a chronic condition involving damage to the intestinal mucosa. IBD includes Crohn’s Disease (CD) and Ulcerative Colitis (UC) [20,21]. These are organic diseases that exhibit dysregulation of the immune system, abnormal inflammatory responses to commensal bacteria, and structural alterations that can be identified through medical examinations and biochemical parameters [22].

Considering the approach presented [2,14,15] and the similar medical symptomatology of the two gastrointestinal diseases, the aim of this scoping review was to systematically identify studies that investigated mentalization in patients with IBD and/or IBS.

## 2. Materials and Methods

### 2.1. Protocol

To collect the findings related to mentalization in patients with IBS or IBD, a scoping review was carried out in line with the Preferred Reporting Items for Systematic Review and Meta-analysis Protocols extension for Scoping Review (PRISMA-ScR) guidelines (checklist in Table S1). A scoping review is methodologically indicated to summarize the results for the constructs of interest in case they were examined using heterogeneous methods and to enable the identification of aspects on which future research should focus [23,24].

In particular, we synthesized the results of mentalization, ToM, metacognition, and RF in patients with IBD and/or IBS, and, if possible, we compared the findings of these populations with those of patients with other conditions or healthy controls. Furthermore, we elucidated the relevance and possible differences in the considered constructs under the functional and organic conditions, thus providing direction for future research.

### 2.2. Eligibility Criteria

The PICOS scheme (participants (P), interventions (I), comparisons (C), outcomes (O), and study designs (S)) was used to further determine the inclusion criteria for this study [25]. Articles were included if they report data on adults with IBS and/or IBD (P), undergoing any type of treatment for these gastrointestinal conditions (I), with or without comparison groups (C) (i.e., healthy controls or patients with other diseases). The outcome considered was the evaluation of mentalization and its implications for mental and physical well-being (O). Studies with cross-sectional and longitudinal designs were both considered for this review (S).

Peer-reviewed research papers (i.e., original articles, brief reports, commentaries, letters to editors, and reviews) published in English were eligible for inclusion. The exclusion criteria were as follows:

- Studies that did not use validated measures to investigate these constructs or that used ad hoc surveys or qualitative methods.
- Papers that did not contain research data or complete information (i.e., case reports, study protocols, perspective articles, and meeting abstracts).
- Papers published but not peer-reviewed (i.e., gray literature) or under review at the time the search was carried out.

### 2.3. Information Sources and Search Strategy

**Figure 1:**
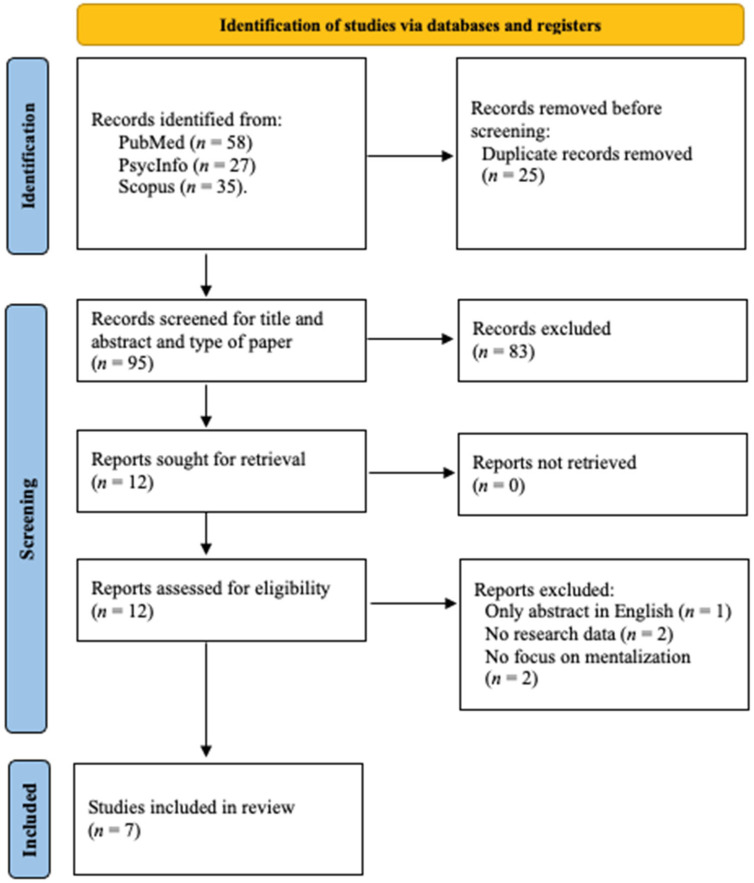
Flow diagram of article selection [24].

A literature search was conducted on 20 January 2023, in the following databases: PubMed, PsycINFO, and Scopus, with the Boolean strings: (“mentaliz*” OR “metacogniti*” OR “theory of mind” OR “ToM” OR “reflective function*”) AND (“irritable bowel syndrome” OR “IBS” OR “inflammatory bowel disease” OR “IBD”). Using this search string, 120 records published between 1988 and 2022 were identified (see Figure 1). Using cross references, no additional articles were found.

### 2.4. Studies Selection 

Studies were selected by three authors (AB, AR, and NS). First, NS skimmed the articles according to their titles and abstracts. Second, AR and NS read the full text of the selected articles.

Following the aforementioned steps, a literature search was performed again by AB to ensure that no records were missed and/or excluded during the selection process.

Disagreements on the inclusion or exclusion of publications were discussed by all authors until an agreement was reached.

### 2.5. Data Extraction

All authors contributed to determining the information extracted from these studies. Two reviewers (AB and NS) tracked the data and discussed the results. Data items extracted from each included study were author(s), year of publication, participants, mean age, instruments used to assess mentalization, other psychological variables measures, and the main results of the studies reviewed.

## 3. Results

A summary of the main characteristics and findings of the seven included studies is provided in Table 1. The selected articles were published between 2019 and 2021. Three of these studies were conducted in Germany, two in Italy, one in Austria, and one in Iran. All articles had a cross-sectional study design.

Among the included studies, three compared patients suffering from IBS with patients diagnosed with IBD [26,27,28], while other studies focused on patients with one of these conditions (IBS or IBD) and compared them with a control group [29,30,31,32].

**Table 1:** A summary of the main characteristics and findings of the included studies (*n* = 7).

Authors (Years)	Participants	Mean (SD) Age	Measures of Mentalization	Measures of Other Psychological Variables	Main Results
Agostini et al. (2019) [29]	96 IBD; 102 HCs.	IBD = 39.6 (14); HCs = 36.3 (12.3)	RFQ; RMET	ASQ	RMET mean (SD) scores were 22.21 (4.38) for IBD and 24.37 (3.17) for HCs. RFQ-Certainty mean (SD) scores were 1.12 (0.99) for IBD and 0.99 (0.63) for HCs. RFQ-Uncertainty mean (SD) scores were 0.72 (0.62) for IBD and 0.59 (0.49) for HCs. No difference was found between the two groups for RFQ. IBD showed lower scores in the RMET (*p* < 0.001) than in HCs.
Berens et al. (2019) [26]	127 IBS; 127 IBD (93 with active symptoms, 34 with non-active symptoms); 127 HCs.	IBS = 36.5 (13.4); IBD = 35.3 (12.3); HCs = 35.1 (13.6)	MZQ	ECR-RD12; SSS-8; PHQ-9; GAD-7; WI-7; ACE	MZQ mean (SD) scores were 35.3 (11.4) for IBS, 32.2 (11.0) for IBD, and 29.4 (10.1) for HCs. IBS reported lower scores of MZQ compared to both HCs (*p* < 0.001) and IBD (*p* = 0.036). IBD showed lower scores of MZQ compared to HCs ( *p* = 0.036). IBS showed lower scores of MZQ compared to IBD without active symptoms (*p* = 0.003,). Whereas, no difference was found between IBS and IBD with active symptoms (*p* = 0.390).
Quattropani et al. (2019) [27]	Study 1: 28 IBS; 33 IBD.	Study 1: IBS = 38.1 (12.2); IBD = 33.9 (11.8)	MCQ-30	ANPS	Study 1: IBD mean (SD) scores were MCQ-POS, 10.97 (4.57); MCQ-NEG, 15.15 (3.85); MCQ-CC,12.06 (3.98); MCQ-NC, 14.15 (3.87); MCQ-CSC, 17.27 (3.20). IBS mean (SD) scores were MCQ-POS, 11.89 (4.57); MCQ-NEG, 12.57 (3.51); MCQ-CC,12.50 (5.25); MCQ-NC, 13.04 (3.72); MCQ-CSC, 17.29 (2.71). No differences were found between the IBD and IBS on MCQ-30 scores.
Quattropani et al. (2019) [27]	Study 2: 20 IBS; 20 UC; 19 CD.	Study 2: IBS = 39.3 (19.8); UC = 37.32 (12.7); CD = 39.8 (14.5)	MCQ-30	ANPS	Study 2: IBS mean (SD) scores were MCQ-POS, 10.40 (4.90); MCQ-NEG, 13.65 (3.73); MCQ-CC,12.25 (4.61); MCQ-NC, 13.15 (3.15); MCQ-CSC, 18.25 (2.45). UC mean (SD) scores were MCQ-POS, 11.50 (4.50); MCQ-NEG, 14.73 (4.29); MCQ-CC,13.05 (5.33); MCQ-NC, 14.45 (4.68); MCQ-CSC, 17.64 (2.70). CD mean (SD) scores were MCQ-POS, 10.68 (4.87); MCQ-NEG, 15.37 (4.92); MCQ-CC,14.47 (5.35); MCQ-NC, 15.42 (4.55); MCQ-CSC, 17.53 (2.74). No differences among the three groups on MCQ-30 scores.
Berens et al. (2021) [28]	199 patients with IBD, IBS, or other DGBI: 92 with SSD, 107 without SSD.	Overall = 37.5 (14.8); with SSD = 38.3 (14.4); without SSD = 36.7 (15.2)	MZQ	ECR-RD12; SSS-8; PHQ-9); GAD-7; WI-7; ACE; Psy-Ba-Do; OPD-SQ; SSD-12	MZQ median (IQR) scores were 2.4 (0.8) for overall, 2.2 (0.8) for patients without SSD, and 2.6 (0.8) for those with SSD, on a range of 1–5. Patients with SSD showed lower MZQ scores than patients without SSD (*p* = 0.001).
Dzirlo et al. (2021) [30]	30 IBS; 32 NAP patients; 28 AD patients.	IBS = 40.8 (11.3); NAP = 38.2 (16.3); AD = 37 (3.8)	RFS (based on BRFI)		RFS mean (SD) scores were 2.7 (1.4) for IBS, 1.4 (1.9) for NAP, and 3.3 (1.7) for AD. Results showed significant differences among the three groups (*p* < 0.001), particularly between IBS and NAP (*p* = 0.008). No differences were found between IBS and AD. RFS mean scores were 2.1 for IBS-M, 3.1 for IBS-D, and 4.4 for IBS-C. There were differences between IBS-M and IBS-C (*p* = 0.023). No significant differences between IBS-M and IBS-D, and between IBS-D and IBS-C.
Engel et al. (2021) [31]	62 IBD: 31 CD; 31 UC; 31 HCs.	CD = 39.2 (14.7); UC = 38.5 (14.0); HCs = 38.5 (13.3)	MZQ	OPD-SQ; ECR-RD12; GAD-7; PHQ-9	MZQ median (IQR) scores were 2.07 (1.00) for CD, 2.13 (1.00) for UC, and 1.73 (1.07) for HCs, on a range of 0–4. No differences were found among the three groups in the MZQ score (*p* = 0.290).
Zargar and Kavoosi (2021) [32]	50 IBS; 50 CHD patients; 50 HCs.	IBS = 35.4 (12.3); CHD = 50.6 (11.6); HCs = 34.5 (11.7)	MCQ-30	ASI	IBS mean (SD) scores were MCQ-POS, 18.05 (3.85); MCQ-NEG, 14.88 (4.39); MCQ-CC,16.18 (4.14); MCQ-NC, 14.40 (3.74); MCQ-CSC, 15.98 (3.90). CHD mean (SD) scores were MCQ-POS, 16.18 (4.32); MCQ-NEG, 13.39 (4.05); MCQ-CC,17.34 (4.60); MCQ-NC, 12.90 (3.25); MCQ-CSC, 14.47 (4.48). HCs mean (SD) scores were MCQ-POS, 15.96 (4.91); MCQ-NEG, 14.48 (4.24); MCQ-CC,15.25 (4.02); MCQ-NC, 13.48 (3.71); MCQ-CSC, 14.96 (4.31). IBS showed higher scores on MCQ-POS than HCs (*p* < 0.05). No significant differences were found between IBS and CHD.

SD: Standard Deviation; IBS: Irritable Bowel Syndrome; IBD: Inflammable Bowel Diseases; HCs: healthy controls; RFQ: Reflective Functioning Questionnaire; RMET: Reading the Mind in the Eyes Test; MZQ: Mentalization Questionnaire; MCQ-30: Metacognitions Questionnaire-30, MCQ-POS: Positive beliefs, MCQ-NEG: Negative beliefs, MCQ-CC: Cognitive Confidence, MCQ-NC: Need to Control thoughts, MCQ-CSC: Cognitive Self-Consciousness; BRFI: Brief Reflective Function Interview; RFS: Reflective Functioning Scale; ASQ: Attachment Style Questionnaire; ECR-RD12: Experiences in Close Relationships Scale; SSS-8: Somatic Symptom Scale; PHQ-9: Patient Health Questionnaire; GAD-7: Generalized Anxiety Disorder questionnaire; WI-7: Whitley Index; ACE: Adverse Childhood Experiences criteria; UC: Ulcerative Colitis; CD: Crohn’s Disease; ANPS: Affective Neuroscience Personality Scale; DGBI: Disorders of Gut–Brain Interaction; SSD: Somatic Symptom Disorder; IQR: interquartile range; OPD-SQ: Operationalized Psychodynamic Diagnosis-Structure Questionnaire; Psy-Ba-Do: Psychosomatic Basis Documentation questionnaire; SSD-12: Somatic Symptom Disorder B criteria scale; NAP: Non-Affective Psychosis; AD: Affective Disorders; IBS-M: Irritable Bowel Syndrom-Mixed; IBS-C: Irritable Bowel Syndrom-Costipation; IBS-D: Irritable Bowel Syndrom-Diarrhea; CHD: Coronary Heart Diseases: ASI: Anxiety Sensitivity Index.

With regard to the instruments used to assess mentalization, three studies used the Mentalization Questionnaire (MZQ) [26,28,31], two studies used the Metacognitions Questionnaire–30 (MCQ-30) [27,32], one study used both the Reflective Functioning Questionnaire (RFQ) and Reading the Mind in the Eyes Test (RMET) [29], and one study used both the Brief Reflective Function Interview (BRFI) and Reflective Functioning Scale (RFS) [30].

Regarding participant characteristics, the mean age of IBD patients involved in the studies was between 33.9 years [26] and 39.6 years [29], while the mean age of IBS patients was between 35.4 years [32] and 40.8 years [30].

Looking at studies in detail, the study by Agostini et al. [29] showed significantly lower RMET scores for patients with IBD than the healthy controls (HCs) (*p* < 0.001); conversely, no significant difference was found for RFQ score (*p* > 0.05). A significant positive correlation was found between RMET scores and one of the subscales of the Attachment Style Questionnaire (ASQ). Moreover, positive correlations were found between a subscale of the RFQ and subscales “need for approval” (r = 0.243, *p* = 0.017) and “preoccupation with relationships” (r = 0.267, *p* = 0.009) of the ASQ. Finally, regarding the association between mentalization and IBD, negative correlations were found between RMET scores and disease activity (r = −0.327, *p* = 0.001) [29].

Another study explored the same dimensions in IBD patients, assessing other variables, such as psychological distress and personality dimensions intended as psychodynamic structural characteristics [31]. Specifically, IBD patients were divided into subgroups: patients with CD, patients with UC, and a group of matched HCs. Regarding mentalization abilities, no differences were found among the three groups in terms of MZQ scores (χ²(2) = 2.475, *p* = 0.290, d = 0.147). If only UC and HCs groups were compared, there was a small difference in the mean rank (UC = 2.13 (1.00) vs. 1.73 (1.07)), although it was not significant (U(1) = 349.500, *p* = 0.139, d = 0.389) [31].

Furthermore, Berens et al. [26] investigated whether patients with IBS showed higher psychological distress and psychological risk factors (i.e., adverse childhood experiences, attachment style, and mentalization) than matched patients with IBD and HCs [26]. Regarding mentalization, the results showed that patients with IBS reported lower MZQ scores than both HCs (z = −4.239, *p* < 0.001, and η^2^ = 0.071) and IBD patients (z = −2.096, *p* = 0.036, and η^2^ = 0.017). Moreover, patients with IBD had lower MZQ scores than HCs (z = −2.117, *p* = 0.036, and η^2^ = 0.018) [26]. Additionally, they explored whether the possible differences between IBS and IBD patients were because of differences in symptom activity. Patients with IBS showed lower MZQ scores than IBD patients without active symptoms (z = −3.380, *p* = 0.003, and η^2^ = 0.071). In contrast, when patients with IBS were compared with IBD patients with active symptoms, no differences were observed in MZQ scores (z = −0.884, *p* = 0.390, and η^2^ = 0.004) [26].

The same research group aimed to clarify the presence of mentalizing deficits in patients with chronic gastrointestinal complaints (IBD, IBS, or other Disorders of Gut–Brain Interaction—DGBI) with or without a Somatic Symptom Disorder (SSD) [28]. The psychological burden due to somatic symptoms was captured by the SSD-B criteria scale (SSD-12; range, 0–48). Based on this criterion, the sample was divided into two subgroups, and several psychological aspects were assessed. Focusing only on mentalizing ability, patients with SSD showed lower MZQ scores than patients without SSD (z = −3.287, *p* = 0.001, and d = 0.5) [28].

Another article compared patients with IBS and IBD and was characterized by two separate studies [27]. In the first study, the aim was to compare metacognitive ability (MCQ-30) and some affect dimensions (Affective Neuroscience Personality Scale—ANPS) between patients with IBS and patients with IBD. Results showed no differences between the two groups in MCQ-30 scores. Moreover, in IBD patients, the “negative beliefs about worry” subscale of the MCQ-30 was significantly and positively correlated with the “fear” (r = 0.72, *p* < 0.01), “anger” (r = 0.66, *p* < 0.01), and “sadness” (r = 0.54, *p* < 0.01) subscales of the ANPS. Moreover, the “anger” subscale of the ANPS was significantly and positively related with the “cognitive confidence” (r = 0.61, *p* < 0.01) and the “need to control thoughts” subscales of the MCQ-30 (r = 0.51, *p* < 0.01). Similarly, in IBS patients, the “negative beliefs about worry” subscale of the MCQ-30 was significantly and positively correlated with the “fear” (r = 0.55, *p* < 0.01), “anger” (r = 0.39, *p* < 0.01), and “sadness” (r = 0.51, *p* < 0.01) subscales of the ANPS [27].

In the second study [27], the sample consisted of three groups: patients with IBS, UC, and CD. The main aim of the study was to explore possible differences in metacognitive abilities among the three groups. In summary, no differences were found among the three groups in MCQ-30 scores [27].

The last two studies evaluated mentalization in patients with IBS compared to patients with other diseases. Zargar and Kavoosi [32] compared patients with IBS, Coronary Heart Disease (CHD), and HCs to investigate metacognitive beliefs and anxiety sensitivity. The study indicated that patients with IBS showed significantly higher scores on the “positive beliefs about worry” subscale of the MCQ-30 than HCs (F = 2.55, *p* < 0.05). No significant differences were found between the IBS and CHD groups in terms of metacognitive beliefs (*p* > 0.05) [32].

Finally, Dzirlo et al. [30] conducted a study among patients with IBS, patients with Non-Affective Psychosis (NAP), and patients with an Affective Disorder (AD) to investigate RF. The results showed lower RF scores in the NAP group than in the IBS group (z = −3.013, *p* = 0.008), while no significant differences were observed between the IBS and AD (z = 0.990, *p* = 0.322) groups. Moreover, the authors aimed to explore possible differences between IBS subgroups (IBS with Diarrhea—IBS-D, Mixed—IBS-M, or with Constipation—IBS-C). Post-hoc tests showed higher RF scores in the IBS-M group than in the IBS-C group (z = −2.669, *p* = 0.023). There were no significant differences between IBS-M and IBS-D or between IBS-D and IBS-C groups [30].

## 4. Discussion

This scoping review was prompted by the idea of answering the following question: are there differences in mentalization between patients who, despite presenting very similar somatic symptoms, differed in the etiological nature of the gastrointestinal disorders from which they suffered?

At a first glance, it would appear not. Going into detail, we could argue that no significant differences in metacognitive ability were found between IBS and IBD patients [27], but if the symptoms were non-active in IBD patients, IBS patients showed more mentalizing deficits than the former [26]. These findings are in line with the evidence that complex gut–brain interactions affect both psychological factors and chronic gastrointestinal symptoms in IBD patients as well as in IBS ones [22]. Although deficits in mentalization are usually considered predictors of functional somatic symptoms, it is plausible that experiencing chronic gastrointestinal symptoms results in stress and, consequently, impairs the ability to mentalize [14].

The significant relevance of symptom severity is also demonstrated by the significant differences in mentalization abilities between IBS subtypes. Patients with IBS-M were more impaired than IBS-C patients in terms of mentalization [30]. The mixed subtype involves alternating constipation and diarrhea, which is likely to involve anxiety related to the presence of both symptoms and the unpredictability of their alternation [18].

In turn, somatic symptoms seem to be associated with greater difficulty in coping with gastrointestinal symptoms [29]. In the context of low mentalization, patients with IBD may use maladaptive coping strategies when faced with disease symptoms and disease-related stress.

Moreover, it would seem that what determines the difference in mentalization abilities is not the different gastrointestinal disorder (IBS vs. IBD) as much as the presence of Somatic Symptoms Disorders (SSD). Indeed, IBS/IBD patients with SSD showed higher mentalization deficits than those without them [28]. This finding is consistent with the notion that the psychological mechanisms in Somatic Symptoms Disorders are the same for patients with functional or organic disorders [33]. It is plausible that difficulties in attributing and interpreting feelings and thoughts are related to a greater psychological burden due to somatic symptoms.

Although psychological distress is more commonly assumed to be a consequence of gastrointestinal symptoms, a large prospective study found evidence of a bidirectional effect of gastrointestinal symptoms activity and psychological distress [34]. IBS symptom activity may also be determined by previous psychological risk factors, such as higher rates of adverse childhood experiences and insecure attachment styles [28,35].

Indeed, deficits in mentalization were associated with insecure attachment styles and negative affects in both IBS and IBD patients [27,29]. These findings are in line with Fonagy’s theoretical model according to which the development of the ability to mentalize occurs within attachment relationships with caregivers in early childhood. This subsequently determines in adults the ability to understand one’s own and others’ mental and affective states [1,4,5,36].

In conclusion, when groups of IBS patients are compared with groups of IBD patients, studies showed impairments in mentalization in both IBD and IBS patients and suggested that low mentalization ability may play a central role in the development of emotional disturbances and maintenance of gastrointestinal symptoms.

When the two gastrointestinal disease groups are compared with a healthy population, the data appear discordant. Indeed, some studies have suggested that both patients with IBS [28,32] and those with IBD [26,29] showed more impaired mentalization abilities than the HCs. Conversely, no differences were found between IBD patients and HCs in other studies [29,31]. One possible explanation is related to the characteristics of the studies, such as the use of different instruments, the small number of subjects recruited, and the recruitment type.

Furthermore, when IBS patients were compared with other clinical conditions (e.g., affective disorders, coronary heart diseases), the findings showed that their metacognitive abilities were similar [30,32].

These findings seem to suggest the non-specificity of mentalization impairment: the transdiagnostic character of this deficit has been largely confirmed by previous studies that have investigated it in mood and psychotic disorders [37,38,39,40], in other diseases such as borderline personality disorder [41,42,43], eating disorders [44,45], somatic symptoms disorder [46], and breast cancer [47]. This non-specificity of the deficit is also supported by Porcelli’s assertions. The common feature of all syndromes—both medical and psychological, in which this deficit occurs—is a type of psychic functioning that can be ascribed to the concept of affective regulation, regardless of an arbitrary distinction between the psychic/functional and somatic/organic [48].

These results should be interpreted with caution since they are based on a few studies that have limitations. First, all studies included in this scoping review employed a cross-sectional design, which did not allow the establishment of any causal direction between mentalization and IBD/IBS outcomes. Second, the use of relatively small samples made generalization of the results difficult, because the population of patients with IBD/IBS may not be widely represented by the samples used in the studies included in the review. Third, the use of different instruments, often self-report, to assess various constructs also related to the field of social cognition (i.e., ToM and metacognition) did not allow for an adequate comparison of the results. In fact, as we attempted to clarify previously, each construct has different peculiarities and only partly overlaps with the specific construct of mentalization. The interchangeable use of terms and instruments, used to investigate other constructs than those for which they were precisely developed, results in limitations to the review itself and to the methodology that could be employed to synthesize the results of the literature in this specific field. Finally, although the included studies investigated other variables, no models were found that related mentalization to psychological and/or clinical variables by attempting to define more explanatory patterns. The heterogeneity of the variables investigated precludes a systematic synthesis of the data in relation to mentalization. In general, the limitations observed suggest the difficulties often involved in the study of complex and interrelated psychological constructs, together with the inherent limitations of the available research methodologies.

Despite these limitations, the present scoping review represents, to the best of our knowledge, the first contribution to summarizing the available evidence on the involvement of mentalization in IBS and IBD.

## 5. Conclusions

The studies included in this review and those available in the literature on other disorders suggest that a deficit in mentalization could be considered a transversal factor, rather than a factor specific to any particular disorder. Given this non-specificity of the deficit, one could hypothesize that mentalization is a type of psychic functioning that can be ascribed to the concept of affective regulation, regardless of an arbitrary distinction between the psychic/functional and somatic/organic. Therefore, it may be useful to consider not the diseases per se, but the profile of individuals at risk of developing mentalization deficits. These considerations are in line with the biopsychosocial model that provides an understanding of the disease that results from a complex interaction of environmental (e.g., diet, early life experiences, and trauma), psychological (e.g., depression, illness anxiety, and somatization), and biological factors (e.g., gut permeability, altered intestinal microbiome, and immune system alteration) with bidirectional interactions of the brain-gut axis.

From a clinical perspective, improved knowledge of mentalization and its association with psychological distress and impaired quality of life in patients with IBD and IBS may provide new scenarios for psychotherapeutic approaches to the treatment of emotional disturbances based on attachment and mentalization theories.

## 6. Future Directions

Future research should implement new directions to clarify the role of mentalization in IBS and IBD patients through longitudinal studies, allowing comparisons with control groups and more complex model analyses. Moreover, future studies should try to have larger samples and use instruments to assess specific construct of mentalization so that results can be adequately compared.

## Data Availability

Data sharing is not applicable to this article as no new data were created or analyzed in this study.

## Supplementary Materials

The following supporting information can be downloaded at: https://www.mdpi.com/article/10.3390/ijerph20237125/s1, Table S1: Preferred Reporting Items for Systematic Review and Meta-Analyses extension for Scoping Review (PRISMA-ScR) Checklist.
